# Supplementary material for: Synergistic Anti-Inflammatory Activity of Lipid-Free Apolipoprotein (apo) A-I and CIGB-258 in Acute-Phase Zebrafish via Stabilization of the apoA-I Structure to Enhance Anti-Glycation and Antioxidant Activities
Source: Int J Mol Sci. 2024 May 20;25(10):5560. doi: 10.3390/ijms25105560 (PMC11121824; doi:10.3390/ijms25105560)
Supplement: Supplementary file 1 [file ijms-25-05560-s001.zip › Supplementary Methods S1.pdf]

Supplementary Methods

Supplementary methods S1:

S1.1: Isolation of Lipoproteins form the Blood

Lipoproteins (LDL and HDL) were extracted from human blood using density-gradient ultracentrifugation [36]. Blood was donated by voluntary participants aged 25±3 years who had fasted for 16 hours in compliance with the Helsinki guidelines and approved by the Institutional Review Board of the Korea National Institute for Bioethics Policy (KoNIBP; authorization number P01-202109-31-009). A 10 mL blood sample was centrifuged at 4,000×g for 20 minutes to obtain serum. Then, 3 mL of serum was layered on a density-gradient mixture of NaCl (1.019<d<1.063) and NaBr (1.063<d<1.225) and subjected to overnight ultracentrifugation (Himac NX, Hitachi, Tokyo, Japan) at 100,000×g. The separated LDL (1.019<d<1.063) and HDL (1.063<d<1.225) were collected from their respective density zones and dialyzed individually using Tris-buffered saline (pH 8.0). The dialyzed LDL and HDL were then stored in a refrigerator for further use.

S1.2: Purification of Human ApoA-I

The apoA-I was extracted from the HDL using the earlier described method [37]. Briefly, 5 mg of HDL was mixed with 1 mL of chloroform and methanol solution (1:1, v/v) and vortexed. The delipidated apoA-I underwent purification through a Superose 6 10/300 GL column (GE Healthcare) using fast protein liquid chromatography, facilitated by an AKTA purifier system (GE Healthcare, Uppsala, Sweden) using 10 mM Tris-HCl/140 mM NaCl (pH 8.0) solvent system. The purity of the separated apoA-I was assessed through SDS-PAGE.

S1.3: Effect of CIGB-258 on the Oxidation of LDL

The ability of apoA-I and CIGB-258 mixture to prevent LDL oxidation was assessed using a thiobarbituric acid reactive substances (TBARS) assay [38] with malondialdehyde (MDA) standard. More oxidized LDL moved faster to the bottom of the gel because of increased negative and apolipoprotein-B (apo-B) fragmentation. The electromobility of each LDL was compared using agarose gel electrophoresis [38]. Briefly, LDL (1 mg/mL) was incubated with CuSO4 (10 μM) for 4 hr at 37°C, in the presence of an apoA-I:CIGB-258 mixture with molar ratios of 1:0, 1:0.1, 1:0.5, and 1:1. Co-treatment of HDL:CIGB-258 during the oxidation of LDL was also assessed using HDL2 (2 mg/mL) and HDL3 (2 mg/mL) with an apoA-I:CIGB-258 mixture with molar ratios of 1:0, 1:0.1, 1:0.5, and 1:1 under the assumption that the molecular weight of the protein was 28 kDa (apoA-I) in HDL.

Subsequently, the ability of the apoA-I:CIGB-258 mixture to prevent LDL oxidation was assessed using a thiobarbituric acid reactive substances (TBARS) assay [38] and agarose gel electrophoresis [39]. After incubation, the samples underwent electrophoresis (0.5% agarose gel at 50 V) to determine the extent of oxidation. The gel was stained with 1.25% Coomassie brilliant blue to analyze the extent of LDL oxidation and apo-B.

S1.4: Paraoxonase Assay

The paraoxonase (PON)-1 activity toward paraoxon was determined by evaluating the hydrolysis of paraoxon into *p*-nitrophenol and diethylphosphate catalyzed by the enzyme [42]. The PON-1 activity was determined by measuring the initial velocity of *p*-nitrophenol production at 37°C, as determined by measuring the absorbance at 415 nm (Bio-Rad iMark microplate reader, Hercules, CA, USA). The same amount of equally diluted HDL_2 and HDL3 (2 mg of protein/mL, 10 μL) was added to 200 μL of the substrate (paraoxon-ethyl, Sigma D-9286; Sigma, St. Louis, MO) and apoA-I:CIGB-258 mixture in a solution containing 90 mM Tris–HCl, 3.6 mM NaCl, and 2 mM CaCl2 (pH 8.5). The PON-1 activity of 1 U/L was defined as 1 mmol of *p*-nitrophenol formed per minute. The molar extinction coefficient of *p*-nitrophenol was 17,000/M/cm.

S1.5: Anti-Glycation Activity of CIGB-258

The antiglycation effect was assessed by incubating a mixture of apoA-I:CIGB-258 at molar ratios of 1:0, 1:0.1, 1:0.5, and 1:1 (apoA-I (85 μg in 340 μL) and CIGB (0.9 μg, 4.5 μg, and 8.9 μg in 60 μL)) in the presence of 200 μM CML (100 μL). Subsequently, 100 μL of a 0.2 M KH_2PO_4/0.02% NaN_3 buffer (pH 7.4) was added, and the content was incubated at 37°C for 72 hr. The anti-glycation effect was evaluated by measuring the fluorescence between the apoA-I alone and apoA-I+CML in the co-presence of various apoA-I:CIGB-258 mixtures at the excitation wavelength (370 nm) and emission wavelength (440 nm) to assess the degree of advanced glycation reactions, as described elsewhere [41]. The movement of Trp during glycation was also assessed by the WMF measurement at excitation = 295 nm and emission = 305-400 nm to avoid Tyr fluorescence, as described previously [40]. Usually, a greater extent of glycation in apoA-I resulted in a larger increase in WMF, a red-shift of WMF, indicating more exposure of Trp to the water phase due to the unfolding of the tertiary structure during glycation.

S1.6: Visualizing Oxidative Stress and Apoptosis in the Embryos

The reactive oxygen species (ROS) and apoptosis in embryos injected with CML and co-treated with apoA-I:CIGB-258, 1:0, 1:0.1, 1:0.5, and 1:1 of molar ratio, were examined using dihydroethidium (DHE) and acridine orange (AO) fluorescent staining, as previously outlined method [45,46]. Briefly, embryos were immersed in 500 μL of DHE (30 μM) and incubated for 30 min in the dark. After two washes with distilled water, the embryos were observed under a fluorescent microscope with an excitation wavelength of 585 nm and an emission wavelength of 615 nm.

Similarly, the embryos were immersed in 500 μL of AO (5 μg/mL) for 30 min, followed by two washes with PBS. Subsequent visualization occurred under fluorescence microscopy at emission and excitation wavelengths of 505 nm and 535 nm, respectively. The fluorescent images of DHE and AO-stained embryos underwent processing for fluorescent intensity quantification using Image J software version 1.53r (http://rsb.info.nih.gov/ij/ accessed on 15 December 2023).

S1.7: ,Liver Histology and Immunohistochemistry

Liver tissue was obtained through surgical extraction from each experimental group and preserved in 10% formalin for 24 hr. After alcohol dehydration, the tissue was embedded in paraffin, and 5 μm thick sections were treated with poly-L-lysine and stained with Hematoxylin

and Eosin (H&E). The morphological changes in the stained tissue sections were examined using an optical microscope (Motic Microscopy PA53MET, Hong Kong, China). The neutrophils in the different H&E-stained areas were quantified by a microscopic examination across various groups. The neutrophil count was assessed in three distinct sections (n=3) for each group, and the results are expressed as the percentage of the neutrophil count in the different groups relative to the 100% neutrophil count in the CML-injected group.

IL-6 production in hepatic tissue was quantified by immunohistochemical staining using a previously established method [17]. Briefly, a 5 μm thick tissue section was exposed to a primary anti-IL-6 antibody (ab9324, Abcam, London, UK). After overnight incubation at 4°C, the tissue section was developed using Envision + system Kits (code 40001, Dako, Denmark) that included a horseradish peroxidase (HRP) conjugated-secondary antibody specific to the primary anti-IL-6 antibody. IL-6 was quantified using Image J software version 1.53r (http://rsb.info.nih.gov/ij/ accessed on 18 September 2023). This involved converting the IL-6-stained area to an RGB stack and applying threshold levels between 25 (lower limit) and 105 (upper limit) to reduce the inclusion of background staining. All the images were processed using the same threshold values, and the resulting percentage area (corresponding to the IL-6-stained area) was determined.

References:

[17] Cho KH, Nam HS, Kim JE, Na HJ, Del Carmen Dominguez-Horta M, Martinez-Donato G. CIGB-258 Exerts Potent Anti-Inflammatory Activity against Carboxymethyllysine-Induced Acute Inflammation in Hyperlipidemic Zebrafish via the Protection of Apolipoprotein A-I. Int J Mol Sci. 2023;24(8):7044. doi: 10.3390/ijms24087044

[36] Havel, R. J.; Eder, H. A.; Bragdon, J. H. The distribution and chemical composition of ultracentrifugally separated lipoproteins in human serum. *J. Clin. Investig.* 1955;34 (9), 1345-1353, doi: 10.1172/JCI103182

[37] Brewer Jr, H. B.; Ronan, R.; Meng, M.; Bishop, C. [10] Isolation and characterization of apolipoproteins AI, A-II, and A-IV. In *Methods in enzymology*, Vol. 128; Elsevier, 1986; pp 223-246, doi: 10.1016/0076-6879(86)28070-2

[38] Blois, M. S. Antioxidant determinations by the use of a stable free radical. *Nature* 1958;181 (4617), 1199-1200

[39] Noble, R. P. Electrophoretic separation of plasma lipoproteins in agarose gel. *Journal of Lipid Research* 1968;*9* (6), 693-700, doi: 10.1016/S0022-2275(20)42680-X

[40] Ramella NA, Rimoldi OJ, Prieto ED, Schinella GR, Sanchez SA, Jaureguiberry MS, Vela ME, Ferreira ST, Tricerri MA. Human apolipoprotein A-I-derived amyloid: its association with atherosclerosis. PLoS One. 2011;6(7):e22532. doi: 10.1371/journal.pone.0022532

[41] McPherson, J. D.; Shilton, B. H.; Walton, D. J. Role of fructose in glycation and cross-linking of proteins. *Biochem.* 1988;*27* (6), 1901-1907, doi: 10.1021/bi00406a016. [42] Markwell, M. A. K.; Haas, S. M.; Bieber, L.; Tolbert, N. A modification of the Lowry procedure to simplify protein determination in membrane and lipoprotein samples. *Anal. Biochem.* 1978;87 (1), 206-210, doi: 10.1016/0003-2697(78)90586-

[45] Owusu-Ansah, E.; Yavari, A.; Mandal, S.; Banerjee, U. Distinct mitochondrial retrograde signals control the G1-S cell cycle checkpoint. *Nat. Genet.* 2008;40 (3), 356-361, doi: 10.1038/ng.2007.50

[46] Hayashi, M.; Sofuni, T.; Ishidate Jr, M. An application of acridine orange, fluorescent staining to the micronucleus test. *Mutat. Res. Lett.* 1983;120 (4), 241-247, doi: 10.1016/0165-7992(83)90096-9
