# Supplementary material for: Synergistic Anti-Inflammatory Activity of Lipid-Free Apolipoprotein (apo) A-I and CIGB-258 in Acute-Phase Zebrafish via Stabilization of the apoA-I Structure to Enhance Anti-Glycation and Antioxidant Activities
Source: Int J Mol Sci. 2024 May 20;25(10):5560. doi: 10.3390/ijms25105560 (PMC11121824; doi:10.3390/ijms25105560)
Supplement: Supplementary file 1 [file ijms-25-05560-s001.zip › Supplementary Results S1.pdf]

Supplementary Results S2:

S2.1. Selection/screening of the selected dose:

The used dose for the microinjection of carboxymethyllysine (CML, 500 ng/10 nL) was selected based on the screening study where different amounts (0-1000 ng CML) were injected in the embryos to assess their effect on the embryo's survivability. At the CML dose (500 ng/10 nL) ~80% mortality of embryos was observed that remains almost constant up to 1000 ng CML/10 nL dose. Based on the dose showing significant toxicity, we have selected the 500 ng CML concentration for further use **(Supplementary Figure S1A)**.

Similarly, distinct concentrations of CIGB-258 (1-10 ng/10 nL) were tested individually or in the presence of CML (500 ng). We observed that the concentration of CIGB-258 up to 10 ng/10 µL has no adverse effect on embryo survivability and development. Even more, the concentration of CIGB-258 $\geq$1ng/10 nL (up to 10 ng) substantially protects embryos against CML-altered survivability and developmental activities. The concentration of CIGB-258 $\geq$1ng/10 µL (up to 10 ng) displayed a similar protective effect against CML-induced toxicity **(Supplementary Figure S1B)**. However, in this study, we explored much lower concentrations of CIGB-258 (i.e., 14 pg, 70 pg, and 143 pg/10 nL), which individually did not demonstrate protective effects on zebrafish embryo survival affected by CML. Yet, when combined with apoA-I (at 1.4 ng/10 nL), these same concentrations (i.e., 14 pg, 70 pg, and 143 pg/10 nL) exhibited significant protective effects.

Similarly, the different doses of the CML (0-1000 µg/10 µL) have been tested to examine their impact on the survivability of adult zebrafish **(Supplementary Figure S1C)**. Finally, 250 µg CML (equivalent to 3 mM CML, considering the average body weight of zebrafish 300 mg) was selected for further study. Likewise, the does response curve was plotted with CIGB-258 as depicted in the **(Supplementary Figure S1D)**

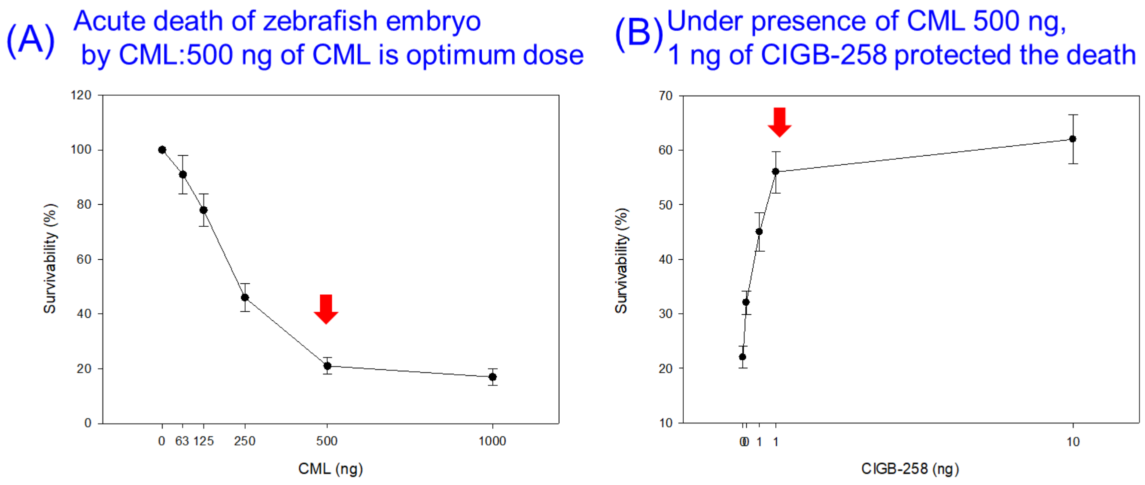

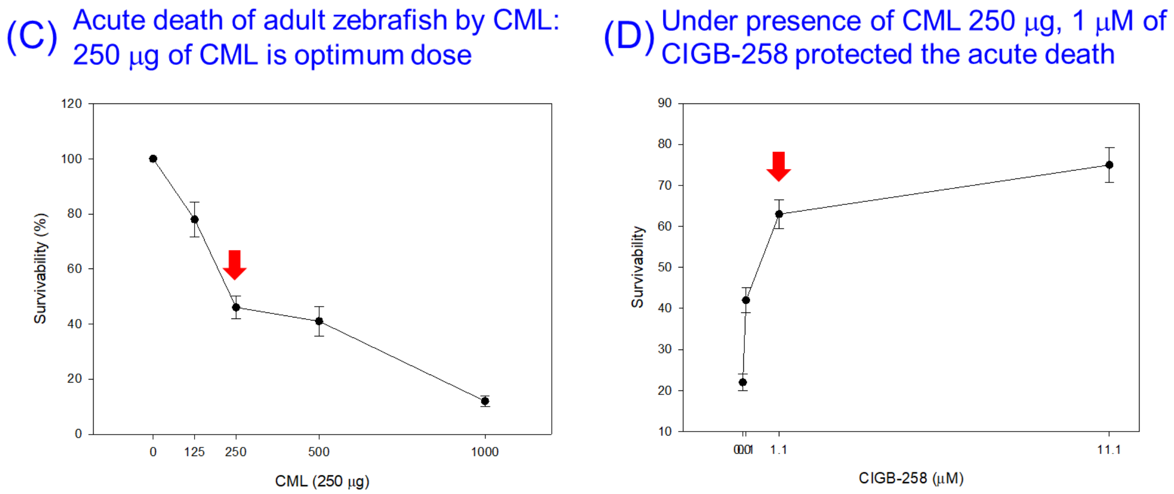

Supplementary Figure S1: (A) Zebrafish embryo survivability in CML:(0-1000 ng); **(B)** Zebrafish embryo survivability under presence of CML 500 ng and varied concentrations (0-10 ng) of CIGB-258**; (C)** Adult zebrafish survivability by CML (0-1000 mg); **(D)** Adult zebrafish survivability under presence of CML 250 mg and varied concentrations of CIGB-258 (0-10 mM).
